# Comparison of long-menu and single-best-answer multiple choice questions in computer-based summative assessments: a randomised controlled trial

**DOI:** 10.1186/s12909-019-1651-6

**Published:** 2019-06-18

**Authors:** Bernard Cerutti, Fabiola Stollar, Monica Escher, Katherine Blondon, Susanne Aujesky, Mathieu Nendaz, Annick Galetto-Lacour

**Affiliations:** 1 Unit of Development and Research in Medical Education, Faculty of medicine, Faculty of medicine, 1 Rue Michel Servet, 1211 Geneva 4, Switzerland; 2 Department of Paediatrics, Children’s Hospital, University Hospitals of Geneva, Geneva, Switzerland; 3 Division of Palliative Medicine, University Hospitals of Geneva, Geneva, Switzerland; 4 Medical Directorate of the University Hospitals of Geneva, Geneva, Switzerland

**Keywords:** Long-menu questions, Computer-based assessment, Computer-based exam, Multiple choice questions, Medical education

## Abstract

**Background:**

Little is known regarding the psychometric properties of computerized long-menu formats in comparison to classic formats. We compared single-best-answer (Type A) and long-menu formats using identical question stems during the computer-based, summative, intermediate clinical-clerkship exams for nine disciplines.

**Methods:**

In this randomised sequential trial, we assigned the examinees for every summative exam to either the Type A or long-menu format (four different experimental questions, otherwise identical). The primary outcome was the power of discrimination. The study was carried out at the Faculty of Medicine, University of Geneva, Switzerland, and included all the students enrolled for the exams that were part of the study. Examinees were surveyed about the long-menu format at the end of the trial.

**Results:**

The trial was stopped for futility (*p* = 0.7948) after 22 exams including 88 experimental items. The long-menu format had a similar discriminatory power but was more difficult than the Type A format (71.45% vs 77.80%; *p* = 0.0001). Over half of the options (54.4%) chosen by the examinees in long-menu formats were not proposed as distractors in the Type A formats. Most examinees agreed that their reasoning strategy was different.

**Conclusions:**

In a non-selected population of examinees taking summative exams, long-menu questions have the same discriminatory power as classic Type A questions, but they are slightly more difficult. They are perceived to be closer to real practice, which could have a positive educational impact. We would recommend their use in the final years of the curriculum, within realistic key-feature problems, to assess clinical reasoning and patient management skills.

## Background

Computer-based assessment facilitates prompt and timely feedback to both students and teachers [1], it is appreciated by the students [2], and broadens the range of item formats compared to a classic paper-based exam with multiple-choice questions. This overcomes, at least partially, the “patients do not present with five choices” criticism [3]. New formats include long-menu questions which are designed to assess decision-making during diagnostic workup, diagnosis and therapy [4]: the computer programme narrows down the potential answers as students type in their free-text responses, limiting the number of options for their final selection. The hidden list of potential answers may be extremely long. For example, the complete international classification of diseases could be used for a question on diagnosis, or an extensive list of active pharmaceutical compounds could be used for a question regarding treatment. The correction and scoring of long-menu questions is faster than for short-answer open-ended questions, which require manual corrections by one or more examiners.

Psychometric properties do not differ significantly between the two formats [5], and more importantly, open-ended format questions are not significantly superior in terms of validity, or in terms of their ability to test higher-order cognitive functioning [6], at least in the context of end-of-education summative assessment. The cueing effect and sheer guessing are decreased when using long-menu questions [7], as students must start typing their answers before having options displayed to choose from.

In addition to the long-menu format, computer-based exams allow the use of other formats, such as key-feature problems where sequential questions need to be answered in a specific order. The sequence mimics real patient management more closely, moving from history-taking to diagnosis and then to treatment. These series of questions can be viewed as simulations [8] falling somewhere between multiple choice questions, which provide an assessment of the proficiency in applying knowledge to descriptions of clinical situations, and examinations with standardised patients, which provide a realistic context for assessing the skills involved in history-taking and performing physical examinations. Combining key-feature problems with long-menu formats seems to be very promising in terms of educational effect, perceived realism and acceptance by the students [9].

In a retrospective study assessing the psychometric performance of 553 items used in 13 computer-based paediatrics exams [10], we found that long-menu questions were easier than the classic single-answer format with five options (difficulty of 81.6% versus 75.7%; *p* = .005) and more discriminating (0.304 versus 0.222; *p* < .001). However, the retrospective observational design was a limitation to this study: since different questions were used in different formats, the contents and underlying learning objectives were likely to have had an impact on both difficulty and discrimination. Furthermore, all the items were related to a single medical speciality.

To the best of our knowledge, little is known about the specific psychometric added value of a long-menu format, compared to a single best answer format, when a teacher chooses how the examinee should answer an exam question: The present study’s main objectives were to compare the level of difficulty and power of discrimination of long-menu questions against single-best-answer multiple choice questions in real-life conditions, i.e. summative undergraduate examinations, by applying both formats simultaneously to the same question stem. The experimental items would thus only differ in the answering modalities: long-menu questions on one hand, and single best answer (best choice), typically from a list of five alternatives (usually called Type A), on the other.

## Methods

This prospective study was carried out at the Faculty of Medicine of the University of Geneva, Switzerland. Apart from the selection exam at the beginning of the curriculum, all the written exams for the medical students have been computer-based since 2011, first using desktop computers and then tablets. We included all the written exams taken during the first and second clinical years of the curriculum (introduction to clinical reasoning, paediatrics, surgery, psychiatry, gynaecology and obstetrics, internal and primary care medicine, intensive and emergency medicine, pathology, ophthalmology, and radiology). In each exam, we identified four Type A questions which could be modified and transformed into long-menu questions (or long-menu questions that could be transformed into Type A questions for the paediatrics exams). For each exam, all enrolled examinees were eligible and randomly divided into two approximately equal sized groups with two different versions of the exam. One group had two questions in the Type A format and the other two questions in the long-menu format, and vice versa for the second group. The question stems for the four questions were kept rigorously identical. The other questions were identical in both versions of the exam, and all questions were included in the computation of the exam score.

The administrative staff randomly assigned (computerized procedure) an examination room seat number to every enrolled examinees. Independently and in parallel, the IT staff randomly uploaded (single randomized allocation) one of the two versions of the exam to every desktop or tablet in the examination room.

We used the Item Management System provided by UCAN (Umbrella Consortium for Assessment Networks, Institute for Communication and Assessment Research, Heidelberg, Germany) combined with CAMPUS (Desktop-based exams) or tEXAM (Tablet-based written exams), which are provided by the same consortium.

### Measures

The primary outcome was the question’s power of discrimination, expressed by the point biserial correlation: this evaluates the item’s ability to differentiate among students on the basis of how well they perform during the exam. It can be viewed as an estimator of the degree to which a single item measures the same underlying construct as all the other items in the exam.

The secondary outcome was the difficulty of the question, defined as the average capacity of students to find the correct answer, i.e. for a single best answer the relative frequency of choice of the unique correct answer. By extension, it was for the long-menu format the relative frequency of choice of the correct answer in the long-menu list (or potential synonyms if some were present in the hidden long-menu list).

For each long-menu question, we determined which distractors the examinees chose. In order to evaluate their perception of the long-menu format, all the examinees received a four-item, online, self-administered questionnaire at the end of the study. The items, measured on a four-point Likert scale (disagree, somewhat disagree, somewhat agree, agree) were: “The long-menu format is more difficult than the other formats”; “My reasoning is different whenever I have to answer a long-menu format”; “The situation I am put in when I have to answer a long-menu format is closer to real life than when I have to answer a classic MCQ item”; and “It is more difficult to answer a long-menu question than an open free-text item. We added the latter item although it is not directly related to the main objective of the study because the long-menu format is sometimes criticized as it does not allow, unlike the open-free text, to validate any chain of characters entered through the keyboard or keypad.”

### Design and sample size

Study design followed a sequential procedure [11]. Indeed, the development of long-menu questions requires a significant amount of work within short timelines. Moreover, two versions of every planned exam were required, which brought an important additional administrative workload. In this specific context, a sequential procedure is particularly useful, as it allows the experimenter to stop a trial earlier while showing clear evidence of either the presence or the absence of a difference between the formats. A list of distractors was developed for each long-menu item. Some lists could be used for several questions.

**Fig. 1:**
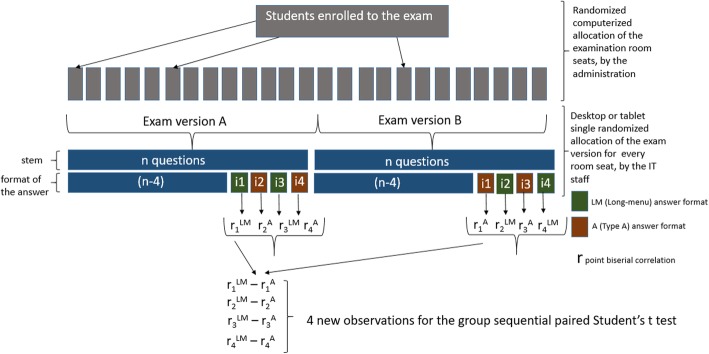
Flow chart of the procedure used for every exam. The labels i1, i2, i3 and i4 stand for the display of the answer for the four experimental item, and the colour stands for the format used

Repeated sequential paired Student’s t-test were performed using for every item the difference between the estimates (point biserial correlation or difficulty) computed from the two versions of the exam (Fig. 1). With a type I error rate of 5% and a type II error rate of 20%, interim analyses after 36, 56, 88 and 112 observations (these numbers were imposed by the organisation of the semestrial exam calendar), using Pocock’s stopping rules [12], would allow us to detect a difference of 0.077 in the point biserial correlation between the Type A and long-menu formats, a difference similar to the one estimated by the retrospective study [10]. In other words, among similar groups of students, within similar exams, for the same question stem, we would expect the discrimination of the long-menu answer format to be 0.077 higher than the discrimination of the type A answer format. We decided to stop the procedure if both the maximum likelihood estimates and the Rao–Blackwell [13] adjusted estimate fell within the defined stopping area boundaries (superiority of the long-menu, superiority of Type A, or futility i.e. equivalence of the two formats). The study started in December 2016 and was expected to end at the latest (due to the sequential design) in January 2019. There were two exam sessions per year: December–January, and May–June. We included in the study all the students enrolled for a given exam, and all the results regarding the four experimental items.

### Analysis

Unless specified, data were summarised as mean ± standard deviation (S.D.). The difficulty and the power of discrimination were provided either by the item management system, or by some in-house validated programs. No manual data handling or computation was required. Student’s t-tests were used to compare two groups of continuous variables. All analyses were run on TIBCO Spotfire S +® 8.1 for Windows (TIBCO Software Inc., Palo Alto, CA, USA), with the additional S + SeqTrial module.

## Results

**Fig. 2:**
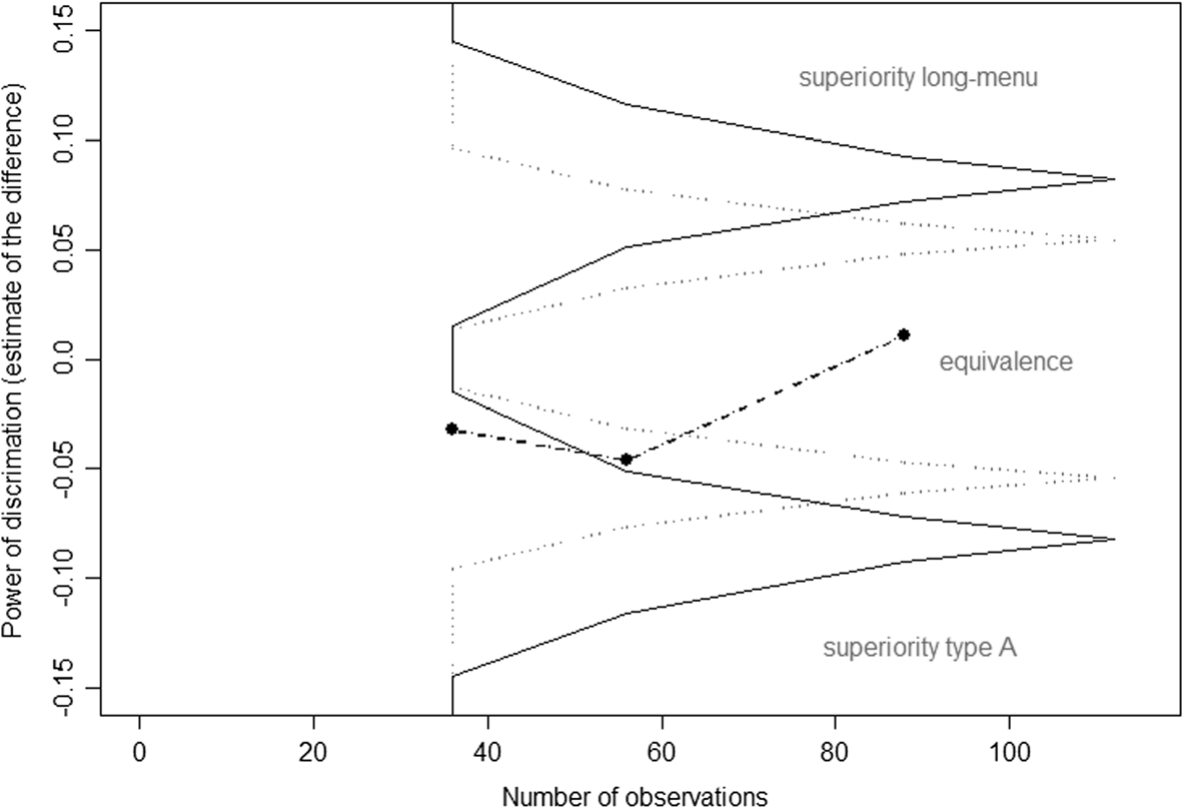
Sequential design interim results and boundaries. Estimates of the differences in discriminatory power between the long-menu and Type A question formats (three points joined with dashed lines) and stopping boundaries of the sequential procedure (solid lines). The dotted lines represent the initial stopping boundaries, and the continuous lines represent the readjusted boundaries at Step 3 (88 observations) taking into account the sequential estimated values of the variability parameters

The exams lasted two academic years, and involved two cohorts of students (*n* = 305; 60% of women; mean age ± S.D.: 24 ± 2.4). The mean total number of items per exam was 58 ± 26, with a mean of 107 ± 42 candidates taking an exam. Most Type A questions (71/88; 81%) included five response options, 14 questions included six options and three included four options. The likelihood estimates had already hit the defined stopping boundaries by the second interim analysis, but the Rao–Blackwell adjusted estimate had not, so it was decided to continue the experiment. The study was stopped after the third interim analysis (88 items from 22 different exams), and we concluded that there was no significant difference regarding the discriminatory powers of the type A and long-menu formats (Fig. 2).

**Fig. 3:**
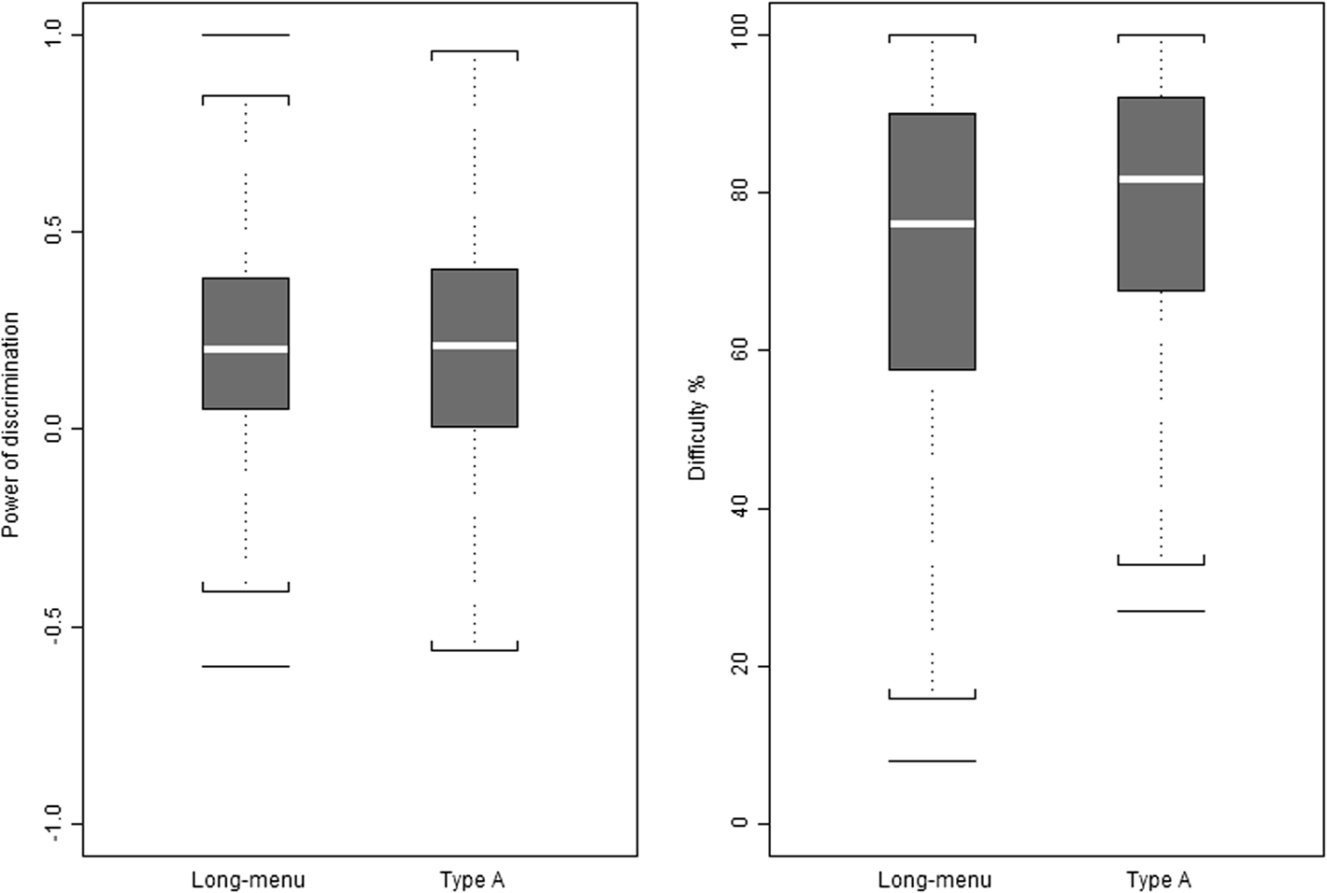
Discriminatory power and difficulty. Discriminatory power (left panel) and difficulty (right panel) of the long-menu and the Type A formats with 88 items

The powers of discrimination and difficulties of both formats are shown in Fig. 3 and reported in Table 1. Although there was no evidence of a difference in the discriminatory powers of long-menu and type A versions of the same question (mean 0.220 vs 0.210; *p* = 0.7948; effect size 0.03), the long-menu versions were more difficult: − 6.34% (average success rate 71.45% vs 77.80%; *p* = 0.0001; 95% confidence interval − 9.45% to − 3.23%; effect size 0.43).

**Table 1:** Discriminatory power and difficulty of the long-menu and Type A formats (*n* = 88 items)

	n	Type A (mean ± S.D.	Long-menu (mean ± S.D.)	Difference between long-menu and type A (95% C.I.)
Discriminatory power^§^	88	0.210 ± 0.273	0.220 ± 0.248	**+ 0.010 (− 0.069 to + 0.091)**
Difficulty^*^	88	77.80% ± 18.83%	71.45% ± 23.29%	**−6.34% (− 9.45% to − 3.23%)**

§ point biserial correlation

* percentage of correct answers

The mean discriminatory power of all the other exam items, i.e. all the items in every exam apart from the four experimental items included either as Type A or long-menu questions, was 0.182 ± 0.202 and showed no evidence of difference with the experimental items (*p*-value Student’s t-test = 0.1688 for the comparison with the long-menu format; = 0.3648 with the Type A format). The average percentage of correct answers was 78.1% ± 20.3%, similar to the Type A experimental items (*p*-value Student’s t-test = 0.8715), but higher than the long-menu experimental items (*p* = 0.010).

Among the options chosen by the students in the long-menu format questions, 616 (45.6%) were distractors listed in the Type A questions, and 736 (54.4%) were not proposed as distractors in the Type A questions.

The results of the survey conducted at the end of the study (n = 184 responders; response rate 60.3%) can be found in Table 2. The main results were the following: “The long-menu format is more difficult than the other formats” (77.5% agreed or strongly agreed); “My reasoning is different whenever I have to answer a long-menu format” (84.8% agreed or strongly agreed); “The situation I am put in when I have to answer a long-menu format is closer to real life than when I have to answer a classic MCQ item” (77.5% agreed or strongly agreed); “It is more difficult to answer a long-menu than an open free-text item” (65.4% disagreed or strongly disagreed).

**Table 2:** Results of the self-administered online questionnaire survey sent to the examinees at the end of the study (*n* = 184 responders)

	n	Disagree			Agree
		1	2	3	4
The long-menu format is more difficult than the other formats	182	2.2%	20.3%	40.4%	34.1%
My reasoning is different whenever I have to answer a long-menu format	184	2.7%	12.5%	49.5%	35.3%
The situation I am put in when I have to answer a long-menu format is closer to real life than when I have to answer a classic MCQ item	182	6.6%	15.9%	47.8%	29.7%
It is more difficult to answer a long-menu than an open free-text item	182	29.1%	36.3%	22.5%	12.1%

## Discussion

To the best of our knowledge, this study is the first randomised controlled trial in real-life conditions of summative assessment to compare long-menu and Type A formats using the same question items. The long-menu format used to record the examinees’ answers had an equivalent discriminatory power to the classic Type A format and it was more difficult. This higher level of difficulty could be explained, in part, by the straightforward mathematical construction of the long-menu format, which reduces the probability of choosing the right answer at random. This was also observed by Schuwirth [7] et al. in their study (average difference in difficulty of 10.7%). Our results were consistent with those observed when using uncued multiple-choice questions [14], which could be considered the paper-based precursor of the computerised long-menu format (average difference of 6.7%).

Our findings did not support the conclusions of our previous retrospective study that had shown a higher discriminatory power for long-menu questions than for other classic formats (e.g. Type A, pick m correct answers from among n options, series of four true or false proposals). The context of our retrospective study was clearly different: psychometric properties were computed from different groups of examinees, and more importantly, the question stems were different, meaning that not only the answer formats were compared but also the topics themselves and the underlying learning objectives. These are likely to be more important elements than the answer format itself [15], both in determining the ratio of correct answers and in stimulating the examinees. Moreover, it is usually considered that a good leading stem for a Type A question should allow the examinee to answer without reading the list of options. Such questions are conceptually closer to long-menu questions, which may help to explain their similar powers of discrimination. Thus, the most plausible explanation of the divergent results between our retrospective study and the current one is that the difference of discriminatory power observed in the retrospective study could not be ascribed to the answering format (Long-menu versus Type A or k’), but to the question stems themselves. In so far the long-menu format presented advantages, it would not be on the pure psychometric level.

It is interesting to note that more than three quarters of the responding examinees thought that the long-menu format was closer to real life. This assertion is supported by the physicians who often face a long list of alternative diagnoses in their daily practice, but it is also asserted by our results: more than half of the incorrect options chosen by students who had the long-menu format were not among the distractors listed in the type A format. This corroborates the finding of Huwendiek et al. [9] that this sort of question provided a greater stimulus for the intense study of clinical reasoning in patient management than did Type A questions.

A vast majority of responding students acknowledged that they had used a different reasoning approach when trying to answer long-menu questions. We may hypothesise that the long-menu format encourages retrieval practice. Therefore, the cognitive effort required by the long-menu format may support mid- and long-term learning and retention of knowledge. However, future research would be needed to address this issue. The students’ perceptions and their observed increased difficulty with long-menu questions also might bring some evidence of a positive educational effect [9].

Almost two thirds of the responding examinees did not find the format more difficult than the free-text open question format, which not only requires a long process of manual marking but is also more liable to a subjective evaluation, and it has never been clearly proven superior to classic multiple choice [6] or long-menu formats [5, 7].

Our study has limitations. It was carried out in a single faculty and the results might not be generalizable to other faculties. However, students’ results at the Swiss Federal Licensing Exam have been similar across all faculties in the past few years, showing that there were no significant differences in teaching at the various faculties of medicine and in the students’ performances. Moreover, we included all the students, hence avoiding the selection bias of studies conducted on volunteer students. The two formats were compared in many different medical specialities, decreasing the likelihood that the results were influenced by topics and content. Another limitation was the choice of exam items for our study. The items were not designed especially for the study: in each exam, with the exception of paediatrics, we identified Type A questions which could be modified into long-menu questions. This could have led to a selection bias based, for example, on our knowledge of existing long-menu lists that could be easily re-used and adapted, or on the fact that we could not make any significant changes to the existing question items. Finally, the short survey was conducted to measure the examinees’ general perception regarding some item formats: this does not constitute evidence about differences in psychometric properties or reasoning processes between the item formats, but may represent interesting topics for future studies.

## Conclusions

They is no evidence that the long-menu questions and Type A question differ regarding the discriminatory power, but the long-menu questions they are slightly more difficult. Long-menu questions are perceived to be closer to real practice, which could have a positive educational impact. Taking into account the increased time required to develop long-menu items when compared to Type A items, we would recommend their use in the final years of the curriculum, within more realistic key-feature problems, which focus on assessing clinical reasoning and patient management skills.

## Data Availability

The datasets generated and analysed during the current study are not publicly available since they deal with real exam data, which are not made publicly available by our Institution, but are available from the corresponding author on reasonable request.
